# Corrigendum to DRESS syndrome without eosinophilia presented with extensive skin rash and acute respiratory failure

**DOI:** 10.1002/ccr3.8979

**Published:** 2024-05-23

Chaisrimaneepan N, Pruneda C, Elmassry M, Abdelnabi M. DRESS syndrome without eosinophilia presented with extensive skin rash and acute respiratory failure. *Clin Case Rep*. 2024;12(5):e8905. Published 2024 May 5. doi:10.1002/ccr3.8905

The first name of the third author is incorrect. It is “Marwan,” but this should be “Marawan.”

We apologize for this error.

